# Antiviral Effect of Matrine against Human Enterovirus 71

**DOI:** 10.3390/molecules170910370

**Published:** 2012-08-29

**Authors:** Yajun Yang, Jinghui Xiu, Xu Zhang, Liangfeng Zhang, Kai Yan, Chuan Qin, Jiangning Liu

**Affiliations:** 1 Key Laboratory of Human Diseases Comparative Medicine, Ministry of Health, Institute of Laboratory Animal Science, Chines Academy of Medical Science & Comparative Medicine Centre, Peking Union Medical College, Beijing 100021, China; 2 Hebei Institute for Food and Drug Control, Shijiazhuang 050011, Hebei, China; 3 Key Laboratory of Human Diseases Animal Models, State Administration of Traditional Chinese Medicine, Institute of Laboratory Animal Science, Chines Academy of Medical Science & Comparative Medicine Centre, Peking Union Medical College, Beijing 100021, China

**Keywords:** enterovirus 71, matrine, antiviral, mouse model

## Abstract

Human enterovirus 71, a member of the *Picornaviridae* family, is one of the major causative agent of hand, foot and mouth disease in children less than six years old. This illness has caused mortalities in large-scale outbreaks in the Asia-Pacific region in recent years. No vaccine or antiviral therapy is available. In this study, antiviral effect of matrine against enterovirus 71 were evaluated *in vitro* and *in vivo*. Matrine could suppress the viral RNA copy number on rhabdomyosarcoma cells. Moreover, matrine treatment of mice challenged with a lethal dose of enterovirus 71 reduced the mortality and relieved clinical symptoms. The results showed that matrine may represent a potential therapeutic agent for enterovirus 71 infection.

## 1. Introduction

Human enterovirus 71 (EV71), a single-stranded, positive-sense RNA virus, belongs to the enterovirus genus of the *Picornaviridae* family. EV71 infections are typically found in children less than six years old, predominantly leading to hand, foot and mouth disease [1,2]. Although this illness is mild and self-limiting in most instances, EV71 infection can also cause severe neurological disorders, such as aseptic meningitis, encephalitis, poliomyelitis-like paralysis, and even death [3]. Since initial identification in 1969 in the United States, severe EV71 outbreaks have been reported periodically throughout the world, particularly in the Asia-Pacific region [4]. Rencently, EV71 epidemics in China have resulted in more than two million HFMD cases and about 1,000 deaths between January 2009 and May 2011 [5]. Unfortunately, no vaccines or antiviral drugs against EV71 have been available for use in the clinic until now [6].

**Figure 1:**
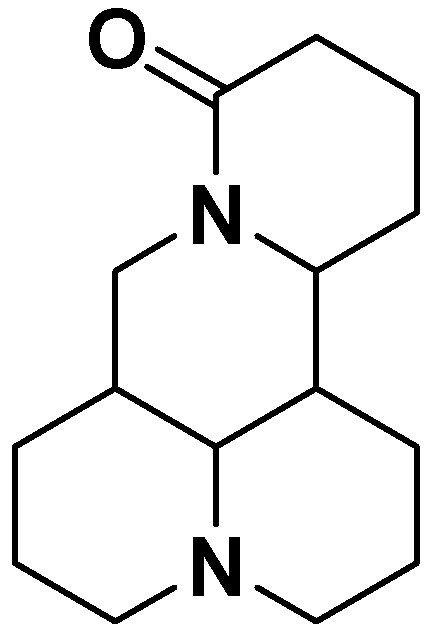
The structure of matrine.

Matrine (Figure 1), a quinolizidine alkaloid, is one of the main active components of the root of Chinese *Sophora* herb plants, including *Sophora flavescens* and *Sophora tonkinensis* [7]. Matrine has been found to possess a variety of pharmacological activities, such as anti-inflammation, anti-tumor, and anti-nociceptive effects. Moreover, matrine has also been used in China for the treatment of viral hepatitis [8]. However, little information is available in the literature about the possible effect of matrine against EV71. In this work, we investigated the antiviral effect of matrine on enterovirus 71 both *in vitro* and *in vivo*.

## 2. Results and Discussion

### 2.1. Determination of the Number of Viral RNA Copies by qRT-PCR in RD Cells

**Figure 2:**
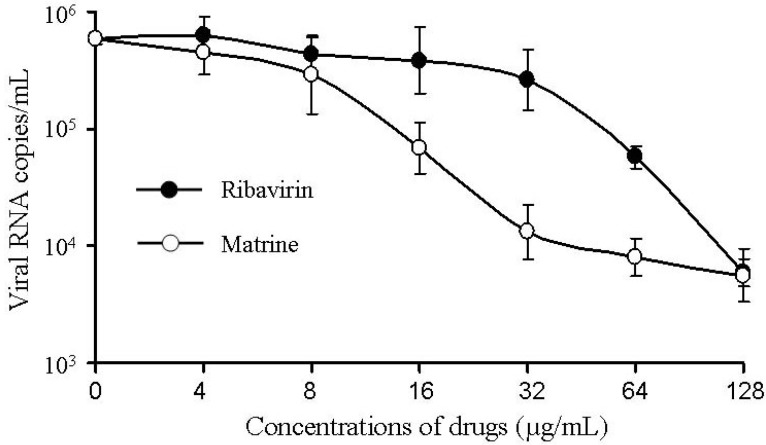
The viral RNA copies in culture supernatant of RD cells treated with matrine at 28 h post infection were detected by qRT-PCR.

The viral RNA copy number detected by quantitative RT-PCR (qRT-PCR) in the infected RD cells were suppressed by matrine in a set of concentrations between 4 and 128 µg/mL, compared to that of the ribavirin control (Figure 2). This observation indicated that matrine had potent antiviral activity in reducing the viral replication in RD cells.

### 2.2. Matrine Reduced the Mortality of Mice upon Lethal EV71 Challenge

**Figure 3:**
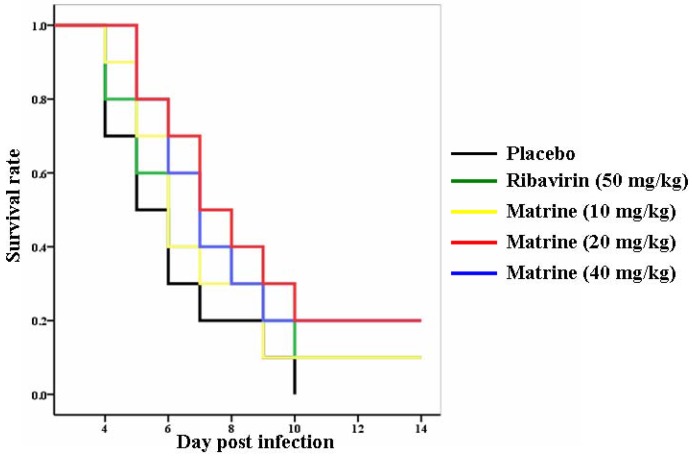
Survival rates of the EV71-infected mice treated with placebo, ribavirin (50 mg/kg) and matrine (10, 20, and 40 mg/kg) were recorded at 14 dpi (n = 30).

The placebo-treated mice developed paralysis at 3 dpi and they all died within 10 dpi. Ribavirin was given at a dose of 50 mg/kg and used as positive control. As shown in Figure 3, treatment with ribavirin enhanced the survival rate of the infected mice nearly to 10%. Matrine proved effective in reducing the mortality rate of the mice when administered at a dose of 10, 20, or 40 mg/kg body weight. At these dosages, 10%, 20% and 20% of the mice were recorded after two weeks, respectively. These results suggested that inhibitory efficacy of matrine was higher than that of ribavirin and 20 mg/kg was a proper dosage to analysis its effect against EV71 in the further experiment.

**Figure 4:**
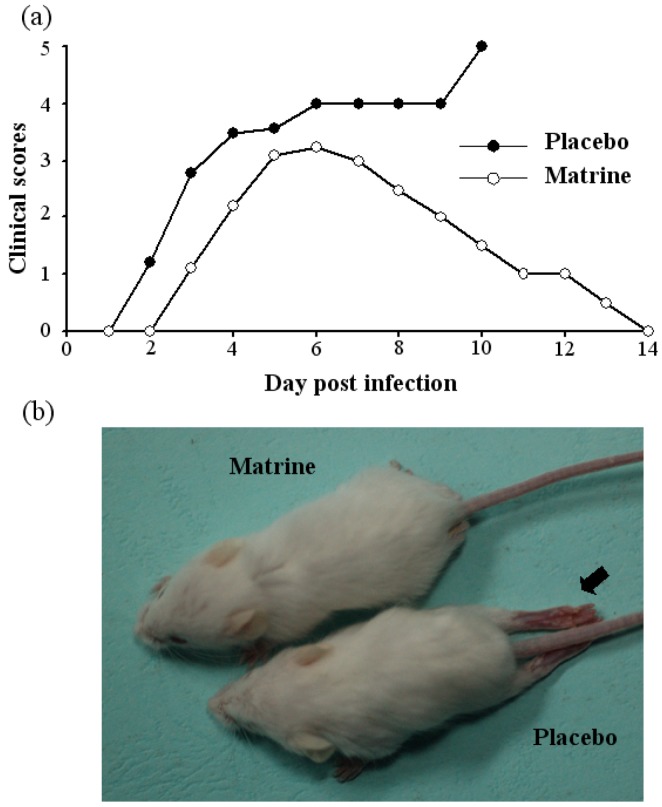
(**a**) The clinical scores of the infected mice treated with placebo or matrine (20 mg/kg) was systematically evaluated in independent experiments (n = 30). (**b**) The typical phenotype of ruffled hair and paralysis of hind limbs caused by EV71 infection at 7 dpi (indicated by arrow) was shown, and the symptoms were prevented in the matrine-treatment group.

**Figure 5:**
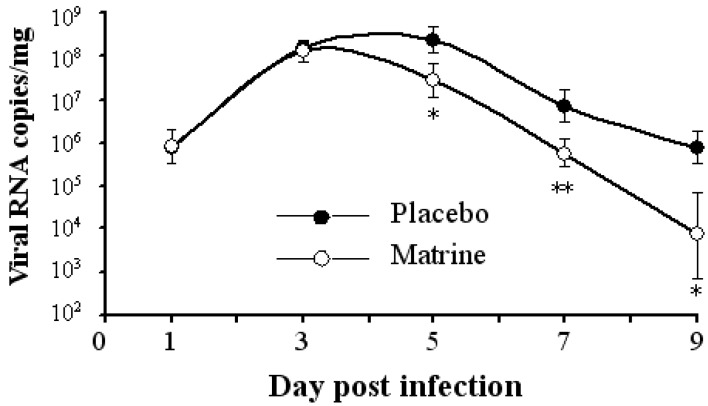
Matrine treatment inhibited the replication of EV71 in muscle tissues of mice (n = 40). The infected mice were treated with the placebo or matrine at a dose of 20 mg/kg. The muscle tissues were sampled and subjected to viral RNA copy analysis by qRT-PCR at 1, 3, 5, 7 and 9 dpi. The data are expressed as the mean values of three independent experiments(*: *p* < 0.05, **: *p* < 0.001).

The clinical scores of infected mice treated with matrine (20 mg/kg) and placebo were subsequently evaluated. Treatment with matrine delayed the paralysis appearance to 1 day later and reduced the clinical scores of the infected mice compared with that of the placebo (Figure 4a). As shown in Figure 4b, the symptoms of the EV71-infected mice were also prevented in the matrine-treatment group. Virus replication of muscle tissues in the mice was significantly decreased by matrine treatment at 5, 7, 9 days after infection detected by qRT-PCR (Figure 5). Moreover, matrine treatment did not cause any obvious side effects in the mice at the tested doses.

In previous studies, treatment with several drug candidates, including ribavirin, bovine lactoferrin, siRNA and type I interferon, were able to enhance the survival rates of infected mice to 18%–100% [9,10,11,12]. However, it is difficult to compare the activity of these drugs because the infection doses of virus and strains were different in these experiments. But we observed that the efficiency of matrine against EV71 infection was better than ribavirin, as shown in evaluation by a mouse model (Figure 3). As we expected, matrine treatment was able to protect the infected-mice from paralysis symptoms by inhibiting EV71 replication (Figure 4b and Figure 5).

In addition, the anti-EV71 efficacy of matrine was tested on RD cells in a plaque reduction assay. It is showed that matrine did not inhibit the cytopathic effect caused by EV71 obviously, but it inhibited the virus RNA copy number in the infected RD cells significantly (Figure 2). Some quinolizidine alkaloids, including matrine and oxymatrine, have been used widely for the treatment of viral hepatitis in china. However, their mode of action against hepatitis virus infection remains unknown. Recently, Gao and co-works discovered that that oxymatrine inhibited hepatitis B virus via down-regulating the expression of heat-stress cognate 70 [13]. Sophoridine, a diastereomer of matrine, was reported to reduce the virus titers in infected myocardial cells. It was also shown that sophoridine significantly increased mRNA expression of IFN-α and IL-10, but decreased TNF-α mRNA expression, suggesting that sophoridine may prevent inflammatory responses and strengthen host resistance against CVB3 [14]. Although the effect against different viruses has been previously reported for matrine, the mechanism was not elucidated. Based on above reports, the anti-EV71 mechanism of matrine might be related to immune regulation. We will focus on an investigation for the anti-EV71 mechanism of matrine in our subsequent experiments.

## 3. Experimental

### 3.1. Reagents

Matrine and ribavirin (purity >98%) were purchased from the National Institute for the Control of Pharmaceutical and Biological Products (China). They were stored at −20 °C in a saline solution before used.

### 3.2. Cells and Viruses

Human RD cells were maintained in Dulbecco’s modified Eagle’s medium (DMEM) containing 10% fetal bovine serum (FBS) as previously described [15]. A clinically isolated EV71 strain FY0805 (GenBank accession No. HQ882182) and the mouse-adapted EV71 strain MP10 (GenBank accession No. HQ712020) derived from FY0805 were cultured in RD cells. The virus titres were determined using a plaque assay as described and working stocks of virus containing 10^9^ TCID_50_/mL were prepared for experiments.

### 3.3. Determination of the Number of Viral RNA Copies by qRT-PCR in RD Cells

For the antiviral assay, RD cells (2 × 10^4^ cells/well) were plated in 96-well plates with DMEM medium lacking antibiotics and grown overnight to 90% confluence at 37 °C. The RD cells were then infected with 100 TCID_50_ of FY0805 and cultured continually in DMEM medium containing 2% FBS. The infected cells treated with matrine in a set of concentrations in saline between 4 and 128 µg/mL were harvested at 28 h post infection to determine the number of viral RNA copies by qRT-PCR.

### 3.4. Mouse Protection Assay

Ten-day-old ICR mice were bred in an AAALAC accredited facility and all of the animal protocols were approved by the Institutional Animal Care and Use Committee of the Institute of Laboratory Animal Science, Peking Union Medical College (GC-09-2077). For lethal EV71 challenge, each ten-day-old mouse was intraperitoneally (i.p.) inoculated with 1 × 10^7^ TCID_50_ (lethal dose) of MP10. At 2 h post infection, the infected mice were injected with different concentrations of matrine in saline once daily for 6 days (i.p.). The placebo group was injected with the same volume of saline as control. The symptoms and survival rates of infected mice were monitored daily for 2 weeks. The clinical scores were graded as follows: 0, healthy; 1, ruffled hair; 2, weakness in hind limbs; 3, paralysis in single hind limb; 4, paralysis in both hind limbs; and 5, death [9]. The muscle tissues of mice were sampled at 1, 3, 5, 7 and 9 dpi for virology analysis.

### 3.5. Determination of the Viral Load

qRT-PCR was used to detect the viral RNA copy number. Briefly, total RNA was isolated from cultured cells or tissues from mice using the TRIzol reagent. The total RNA was then reverse transcribed using random hexamers with a reverse-transcription kit (Promega). The cDNA was subjected to quantitative PCR (QuantiTect SYBR Green RT-PCR kit, QIAGEN) with a Roche LightCycler 3.5 system for 40 cycles. The primers were EV71-S1 (5’-AGATAGGGTGGCAGATGTAATTGAAAG-3’) and EV71-A1 (5’-TAGCATTTGATGATGCTCCAATTTCAG-3’). A fragment corresponding to nucleotides 2462-2635 of FY0805 was adjusted to a concentration gradient (1 × 10^1^ copies/μL to 1 × 10^8^ copies/μL) and was used as standard to calculate the copy number of viral RNA.

### 3.6. Statistics

All data are expressed as the mean ± S.D. The statistical significance of differences in mean values was assessed by Duncan’s multiple-range test following a one-way analysis of variance (ANOVA), and survival rates were analysed by Kaplan-Meier analysis. A *p* value of <0.05 was considered to be significant.

## 4. Conclusions

In conclution, matrine could inhibit EV71 infection in RD cells and reduce the mortality of mice upon lethal EV71 challenge. To the best of our knowledge, this is the first report on the anti-EV71 activity for matrine. The potent anti-EV71 acvivity of matrine makes it the lead candidate for development as antiviral drugs. Although previous studies focused on the structure modification of matrine and synthesized a series of derivatives and evaluated their biological activities, all of derivatives have not critically evaluated EV71 activity [16]. Further studies will be required to determine structure-activity relationships of matrine derivatives and to investigate therapeutic effect of related quinolizidine alkaloids for EV71 infection.
